# Supplementary figures and images for: UID-Dual Transcriptome Sequencing Analysis of the Molecular Interactions between Streptococcus agalactiae ATCC 27956 and Mammary Epithelial Cells
Source: Animals (Basel). 2024 Sep 5;14(17):2587. doi: 10.3390/ani14172587 (PMC11393856; doi:10.3390/ani14172587)

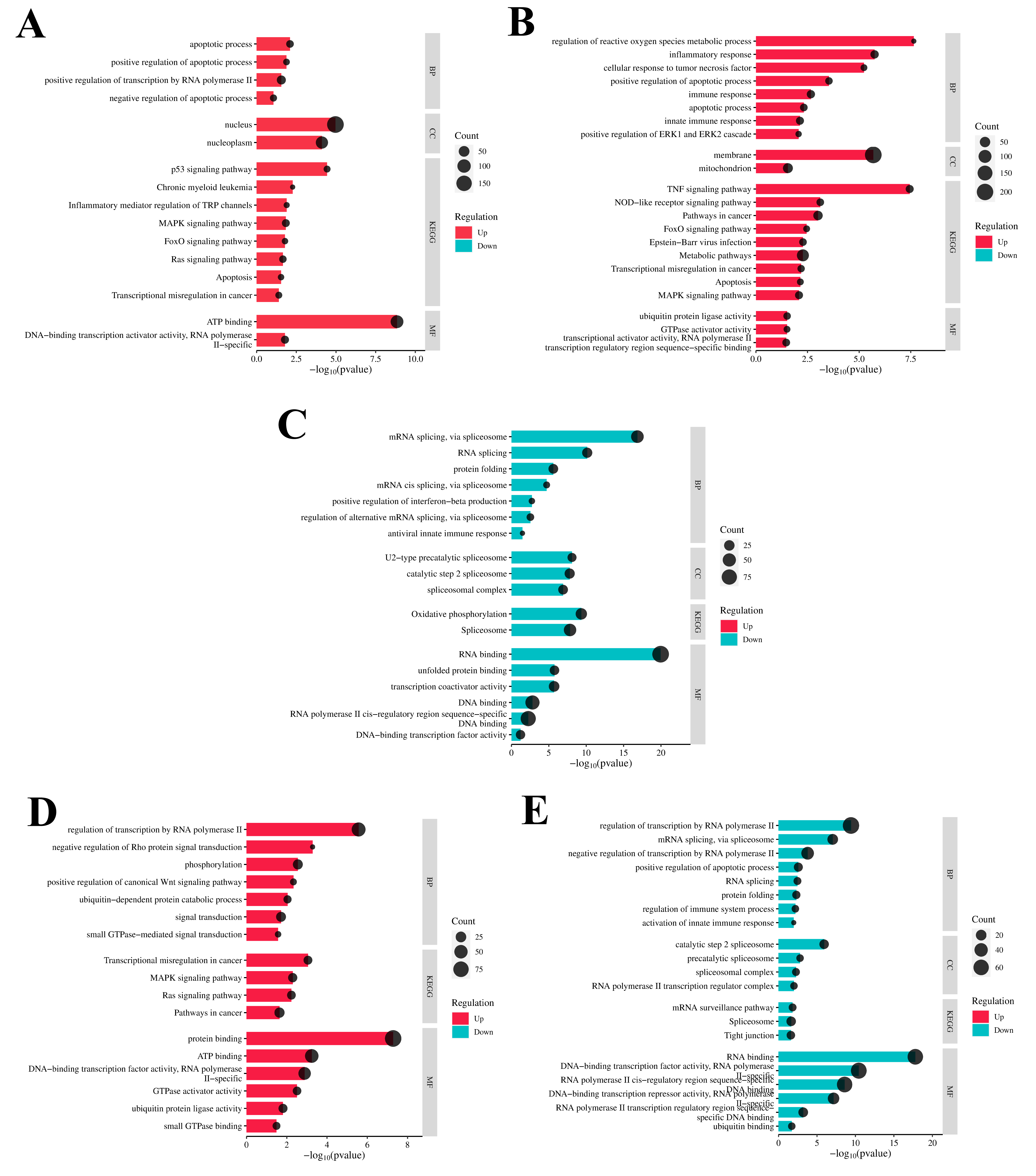

Supplement: Supplementary file 1 [file animals-14-02587-s001.zip › Supplementary Figure S1.tif]
